# Fertility matters: linking male sterility to starch synthesis in *Salvia miltiorrhiza*

**DOI:** 10.1093/plphys/kiag530

**Published:** 2026-07-21

**Authors:** Rose McNelly

**Affiliations:** Assistant Features Editor, Plant Physiology, American Society of Plant Biologists, Rockville, MD, United States; John Innes Centre, Norwich Research Park, Norwich NR4 7UH, United Kingdom

*Salvia miltiorrhiza*, also known as red sage, is a member of the mint family and has been used for treating cardiovascular and cerebrovascular diseases for over 2,000 years (Wang et al 2017). Demand for *S. miltiorrhiza* is outstripping supply due to its long growth period (Shi et al 2020) and the poor fertility of some populations (Liao et al 2023). Fertility in *S. miltiorrhiza* is poorly understood, but a better understanding could allow breeding for increased fecundity and the development of male sterile lines for use in heterosis systems.

Male sterile *S. miltiorrhiza* plants were first identified in 2002 (Shu et al 2012), yet mechanisms causing male sterility have not been fully elucidated. Previously, Liao et al (2023) described a male sterile line from Sichuan (SC) with a significant reduction in pollen starch content. In other plants, including *Arabidopsis* and rice, starch is linked to male fertility as it is crucial for pollen maturity and pollen tube germination (Liu et al 2021). Therefore, the reduction in starch in SC plants could be contributing to male sterility. In a recent *Plant Physiology* article, Shang et al (2026) investigated pollen starch metabolism in SC lines.

Through comparison of anther gene expression in the SC line with a fertile *S. miltiorrhiza* line from Shandong (SD), the authors identified sucrose synthase 1 (*SUS1*) as a candidate for influencing male sterility. SUS1 was upregulated in fertile SD lines in a similar pattern to starch accumulation and can catalyze the interconversion of UDP-glucose and fructose to sucrose, which are key precursors for starch biosynthesis (Stein and Granot 2019). To explore the role of *SUS1* in male sterility, the authors looked for differences in the gene and protein sequences between the sterile SC and fertile SD lines. No differences were found in the SUS1 amino acid sequence, but *SUS1* was more highly expressed in the fertile SD line than the sterile SC line, suggesting that *SUS1* expression may be linked to fertility.

A strength of this article is the suite of overexpression and knockout lines in *S. miltiorrhiza* that were generated to study SUS1. One example is constitutive expression of *SUS1* in the sterile SC background, which significantly increased pollen vitality, although it remained lower than in SD lines. Closer examination of starch in the overexpression lines showed an increase in starch granule number, but not starch granule diameter, and an elevated amylopectin content—how it causes these effects is unclear, as expression of genes linked to granule initiation and amylopectin synthesis were not studied. In a complementary experiment, *SUS1* was knocked out in the normally fertile SD line and caused a significant reduction in pollen vitality and number of seeds produced. Yet, fertility was not zero, insinuating potential redundancy within the *SUS* family. Surprisingly, overexpression of *SUS1* in the normally fertile SD line had no effect, suggesting other factors limit fertility. One such factor could be substrate supply; increasing carbon flux into anthers in SD lines overexpressing *SUS1* could reveal whether substrate supply is the limiting factor. Together, these knockout and overexpression experiments provide robust evidence for SUS1 being a key regulator of male sterility in *S. miltiorrhiza.*

Next, the authors explored regulation of *SUS1*. Using weighted gene coexpression network analysis, they identified 2 transcription factors: *SmWLIM1* (a widely expressed LIM-domain protein 1) and *SmMYB62*. Both were upregulated in fertile SD lines and independently bind the *SUS1* promoter in yeast one-hybrid and dual luciferase assays, although no interactions between them were investigated. Constitutive overexpression of either transcription factor in the normally sterile SC lines increased pollen fertility and germination rate.

While regulation of *SUS1* had been successfully elucidated, the authors were left with the following question—how are *WLIM1* and *MYB62* upregulated in SD plants? One avenue of research was investigating the role of jasmonic acid (JA), as it has links to fertility in *Arabidopsis* and rice (Fartyal et al 2025). Both the *WLIM1* and *MYB62* promoters contain methyl jasmonate (MeJA) responsive elements, and there were higher JA levels in SC anthers compared to SD anthers, suggesting that JA may repress expression of *WLIM1* and *MYB62*. Curiously, *SmWLIM1* and *SmMYB62* had different responses to MeJA. Exogenous application of MeJA to SD anthers reduced *SmWLIM1* and *SUS1* expression, causing a lower starch content, in agreement with JA having a repressive role. Meanwhile, MeJA application upregulated *SmMYB62* at the young microspore stage but downregulated *SmMYB62* at the mature pollen stage, suggesting a more complex JA-independent mode of regulation.

In summary, Shang et al (2026) describe a transcriptional module influencing fertility in *S. miltiorrhiza* whereby JA represses *SmWLIM1* expression, leading to less *SUS1* expression, reduced starch content, and less fertile pollen (Fig. 1). They identified a second transcription factor, *SmMYB62*, which also activates *SUS1* but is mostly JA-independent. The identification of the module raises several interesting questions. For example, is the module conserved in other species? High conservation seems unlikely in *Arabidopsis* as *SUS* is not involved in pollen fertility (Barratt et al 2009) and the *Arabidopsis* homologs of *SmMYB62* and *SmWLIM1* have no known links to pollen starch metabolism. In that case, is it a species-specific fertility module, and what advantages might it have for *S. miltiorrhiza*? Beyond this, can the module be manipulated to increase fertility further, or is it limited by substrate supply? Finally, can we knock out elements in the module to generate male sterile plants? And could these male sterile plants be useful in heterosis-based systems to generate high-yielding *S. miltiorrhiza* plants to meet demand for their beneficial medicinal compounds?

**Figure 1:**
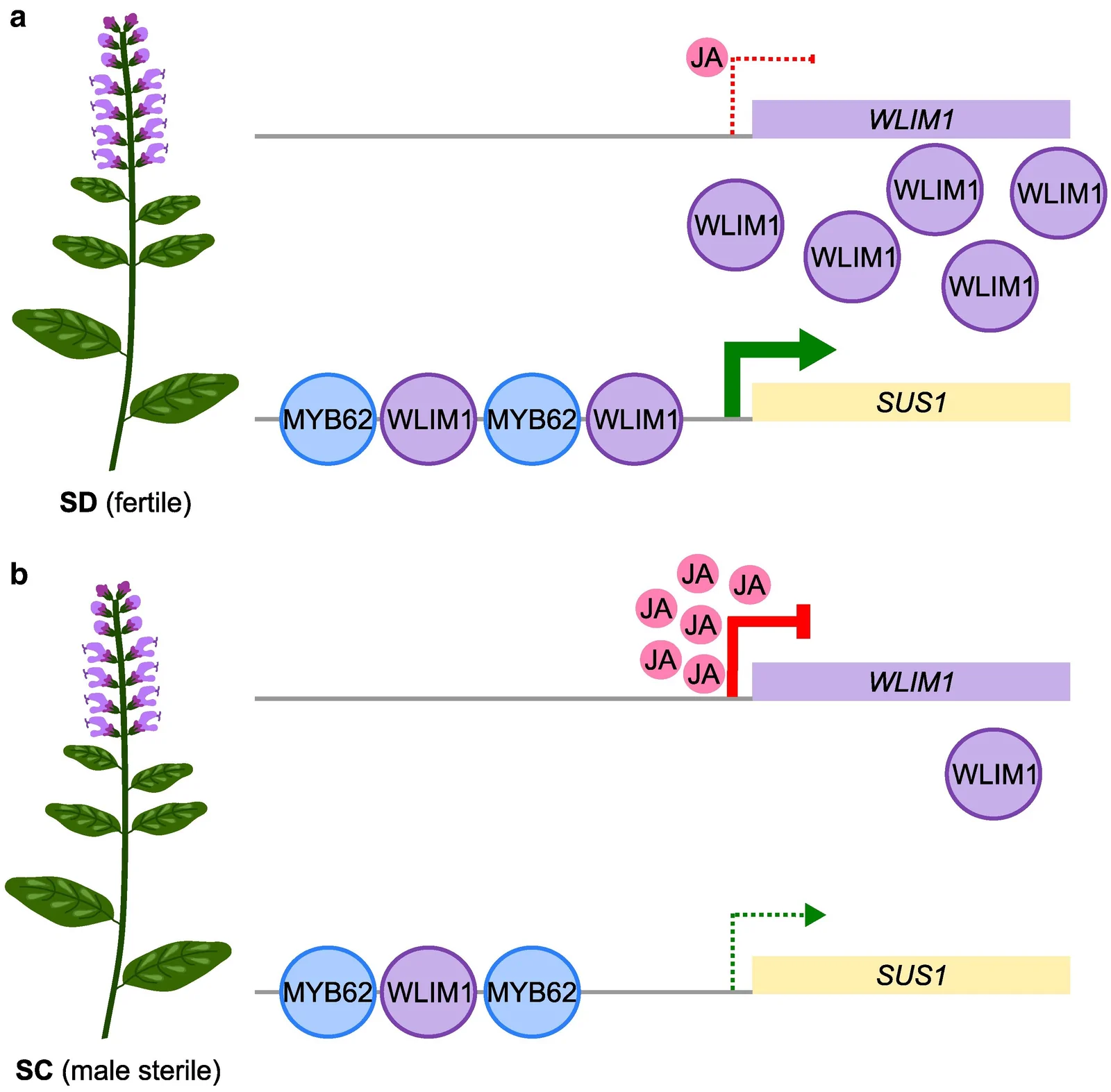
A transcriptional module regulates *SUS1* expression and fertility in *S. miltiorrhiza.* a) In the *S. miltiorrhiza* fertile SD line, *WLIM1* expression is not repressed by JA. WLIM1 binds the *SUS1* promoter alongside MYB62 to promote *SUS1* expression. b) In the *S. miltiorrhiza* male sterile SC line, there are elevated JA levels in anthers. JA represses *WLIM1* expression, so less WLIM1 binds the *SUS1* promoter, and *SUS1* expression is reduced. In both a) and b), jasmonic acid is represented by pink circles, MYB62 by blue circles, and WLIM1 by purple circles. This figure was prepared using Inkscape.

**Recent related articles in *Plant Physiology*:**

- Lv et al (2025) investigated drought responses in *S. miltiorrhiza*. They identified a transcription factor that is upregulated in response to JA and also triggers JA biosynthesis.
- Du et al (2025) identified a genic male sterile pepper (*Capsicum annuum*) and attributed sterility to nonsense mutation in a cytochrome P450 gene.

## Data Availability

No new data were generated or analysed in support of this research.
